# The Cincinnati Lancet and Observer

**Published:** 1878-08

**Authors:** 


The *Cincinnati Lancet and Observer*, and *The Clinic* have consolidated, and will hereafter appear as a weekly under the name of the CINCINNATI LANCET AND CLINIC. Drs. J, C. Culbertson and J. G. Hyndman are editors, and under such able management success is assured to the new enterprise. The first number is well printed, on good paper, filled with readable matter. Each number will consist of sixteen pages, published every Saturday, at $3 50 per annum.

